# Phylogenetic analysis of Helicobacter pylori cagA gene of Turkish isolates and the association with gastric pathology

**DOI:** 10.1186/1757-4749-5-33

**Published:** 2013-11-18

**Authors:** Barik A Salih, Bora Kazim Bolek, Mehmet Taha Yildiz, Soykan Arikan

**Affiliations:** 1 Faculty of Science and Literature, Department of Biology, Fatih University, Istanbul, Turkey; 2 Istanbul Esenyurt University, Vocational High School of Health Services, Program of Medical Laboratory Techniques, Istanbul, Turkey; 3 Department of Surgery, Istanbul Teaching and Research Hospital, Istanbul, Turkey

**Keywords:** *Helicobacter pylori*, Phylogenetic analysis, cagA

## Abstract

**Background:**

The cagA gene is one of the important virulence factors of *Helicobacter pylori*. The diversity of cagA 5′ conserved region is thought to reflect the phylogenetic relationships between different *H. pylori* isolates and their association with peptic ulceration. Significant geographical differences among isolates have been reported. The aim of this study is to compare Turkish *H. pylori* isolates with isolates from different geographical locations and to correlate the association with peptic ulceration.

**Methods:**

Total of 52 isolates of which 19 were Turkish and 33 from other geographic locations were studied. Gastric antral biopsies collected from 19 Turkish patients (Gastritis = 12, ulcer = 7) were used to amplify the cagA 5′ region by PCR then followed by DNA sequencing.

**Results:**

The phylogenetic tree displayed 3 groups: A) a mix of 2 sub-groups “Asian” and “African/Anatolian/Asian/European”, B) “Anatolian/European” and C) “American-Indian”. Turkish *H. pylori* isolates clustered in the mixed sub-group A were mostly from gastritis patients while those clustered in group B were from peptic ulcer patients. A phylogenetic tree constructed for our Turkish isolates detected distinctive features among those from gastritis and ulcer patients. We have found that 2/3 of the gastritis isolates were clustered alone while 1/3 was clustered together with the ulcer isolates. Several amino acids were found to be shared between the later groups but not with the first group of gastritis.

**Conclusions:**

This study provided an additional insight into the profile of our cagA gene which implies a relationship in geographic locations of the isolates.

## Background

*Helicobacter pylori* possess several virulence factors that play important role in the development of gastric diseases. The cag pathogenicity island (cagPAI) a 40-kb locus contains 31 genes among which the cytotoxin-associated gene (cagA) was found to exert a severe damaging effect [1,2]. Several studies showed that cagA-positive *H. pylori* strains are more often isolated from patients with gastric ulcers (GU), duodenal ulcers (DU) and gastric cancer (GC) than those with gastritis (G) [3,4]. Also studies have revealed that the prevalence of cagA-positive strains varies among different populations. In East Asia, nearly all isolated strains were cagA-positive whereas in Western countries this frequency is much lower [5,6].

The structure of the cagA gene reveals a 5′ highly conserved region and a 3′ variable region. The variation in the size of the CagA protein has been correlated with the varying number of repeat sequences located in the 3′ variable region of the gene that encodes the EPIYA motifs [7]. Based on the types of these motifs the CagA proteins were divided into Western and East Asian types. In a previous study it has been shown that there is less than 53% homology between the CagA repeat sequences of Western and East Asian strains indicating the existence of gene variability that might affect the toxin strength [8].

*H. pylori* genetic diversity appears to be a reflection of evolution through thousands of years even before migration out of Africa that lead to geographic spread throughout the world. cagA gene diversity among strains from different geographic regions has been analyzed using different approaches. In one approach EPIYA motifs were used to determine sequence diversity while in others phylogenetic analysis were undertaken on larger portions of the cagA gene [9]. Kawai et al. [10] recently compared the genome sequence of 4 Japanese *H. pylori* strains to the available complete genome sequences of Korean, Amerind, European, and West African strains. They reported differences in gene sequences of virulent factors of the Japanese and Korean strains from the others. Camorlinga-Ponce et al. [11] did phylogenetic analyses of the cagA 3′ region of isolates from various geographic locations and reported that although most *H. pylori* isolates from indigenous communities of Mexico were of the Western type, a new Amerindian cluster neither Western nor Asian was found. Mane et al. [12] also studied the cagA gene of a strain isolated from an Amerindian subject and reported that there was a substantial divergence of that Amerindian strain from the Old World strains. Recently Hirai et al. [13] studied the cagA 3′ region of isolates from asymptomatic healthy Japanese and Thai subjects and compared with those from patients with GC. They suggested that cagA sequence differences between those subjects might be linked to GC incidence in Japan and Thailand. Another study showed that the origin of *H. pylori* isolates from GC patients were different from those with duodenal ulcer in Japan based on a full length cagA sequences analysis [14]. It has been also shown that differences in the prevalence or expression of cagA gene play an important role in the etiology of these diseases [15]. It is also thought that the diversity of cagA 5′ conserved region reflects the phylogenetic relationships among *H. pylori* isolates and their association with the clinical outcome [9-11]. Therefore a comparative analysis of *H. pylori* cagA gene among different isolates may shed light on these concerns. In addition such a study dealing with such analysis of cagA gene is still lacking in Turkey. In this study the diversity of *H. pylori* cagA gene of isolates from Turkish dyspeptic patients was investigated using DNA sequence analysis and a correlation with the clinical outcome was established. We have also conducted a comparative phylogenetic analysis between the cagA 5′ conserved region of our isolates and isolates from different geographic locations of the world to determine the clustering of these isolates within different regions.

## Results and discussion

**Figure 1:**
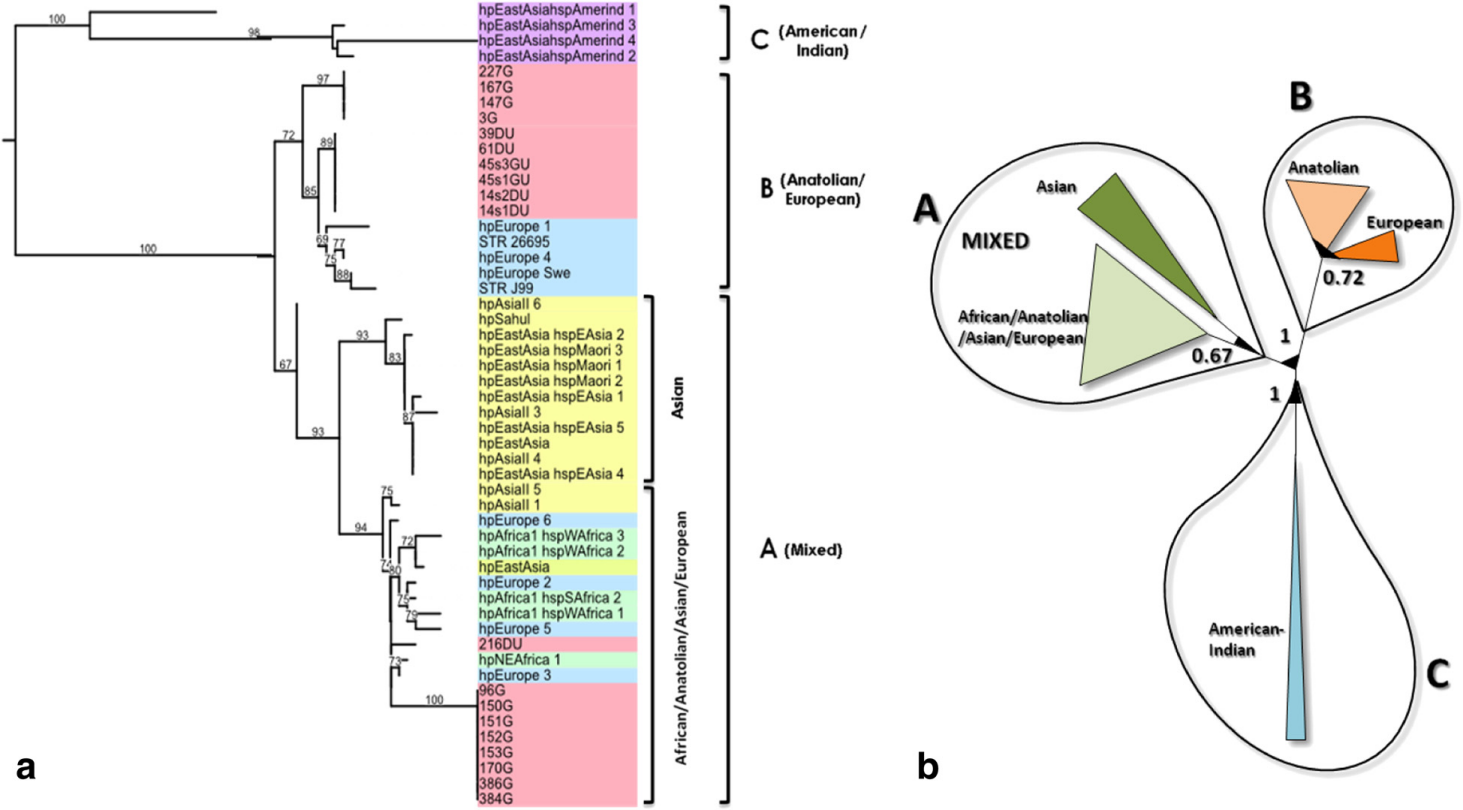
**Maximum likelihood tree of cagA 5′ conserved regions of *H. pylori *isolates from different geographic locations. a)** Tree representation of the Maximum likelihood tree. Colors represent groups of isolates from; Anatolia (Turkish) (pink), Europe (blue), Asia (yellow), Africa (green), American Indian (purple). STR: reference sequences of 26995 and J99 isolates. Numerical on nodes represents bootstrap values. G: Gastritis, GU: Gastric ulcer, DU: Duodenal ulcer. **b)** Graphic representation of the Maximum likelihood tree. Numerical on nodes represents bootstrap values.

*H. pylori* colonies were identified by urease test, gram stain and PCR. A single colony was picked, subcultured and then genomic DNA was isolated. Amplification of the cagA 5′ region revealed a 350 bp band on agarose gel. DNA sequencing of the amplified product was done and the amino acid sequence was deduced. Our constructed phylogenetic tree (Figure 1a) made it possible to distinguish 3 general groups; A, B and C. Group A is a mixed group that contained isolates from nearly all geographic regions and it composed of 2 sub-groups one designated as “Asian” as it contained only isolates from Asia and the other one as “African/Anatolian/Asian/European” as it contained isolates from all those regions. Group B contained isolates from the Anatolian and European region only including the 2 reference strains STR_26695 (gi|306479659|) and STR_J99 (gi|4155035|). Group C is positioned like an out-group with 4 isolates of American-Indian origin. We have found that our Turkish *H. pylori* isolates from the Anatolian region were clustered in 2 locations on the tree as being part of both group A and B. Most of our isolates from patients with gastritis were clustered in the sub-group A (African/Anatolian/Asian/European) but still others were gathered in sub-group B (Anatolian/European). However isolates from DU and GU patients with one exception (strain 216DU) were clustered only in group B (Anatolian/European). Figure 1b delineate those groups in a more visual solid matrix as an unrooted form of the constructed tree. In this figure the bootstrap values represents the reliability of the tree topology.

**Figure 2:**
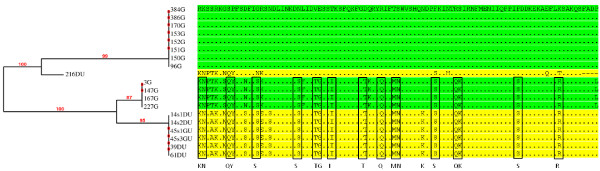
**Maximum likelihood tree of cagA 5′ conserved region sequences of our Turkish isolates.** Specific conserved regions of duodenal ulcer (DU) and gastric ulcer (GU) sequences are highlighted with yellow while gastritis (G) sequences are green. Longitudinal boxes include amino acids shared between isolates from G and DU patients. Dots represent conserved amino acids while numerical on nodes represents bootstrap values.

**Figure 3:**
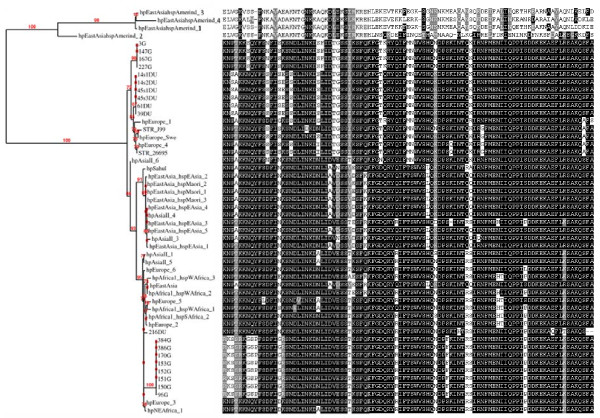
**Maximum likelihood tree of cagA 5′ conserved region of *H. pylori *isolates from different geographic locations and relevant alignment of the sequences.** Bootstrap values are indicated on the tree branches.

We have also constructed a phylogenetic tree only for our Turkish isolates using the same settings as for the whole tree to detect any distinctive features among those from gastritis and ulcer patients (Figure 2). The results showed that isolates from those patients were divergent. We have found that 2/3 of our isolates from gastritis patients were clustered alone while the other 1/3 was clustered together with those isolates from peptic ulcer patients. One interesting finding was the detection of some amino acids that was shared between the later 2 groups (1/3 gastritis and peptic ulcer) but not with the first group of gastritis (longitudinal boxes). Such finding can be further investigated using large number of isolates with known gastric pathology. We also found that some amino acids were unique to the ulcer isolates but not found in the gastritis isolates. Figure 3 depicts a unified origin for the majority of isolates studied that were divergent from the Amerindian isolates which was clearly shown in the alignment set up.

*H. pylori* possess an enormous genomic diversity that facilitates host adaptation [16,17]. The high prevalence of such diversity exerted an impact on the clinical outcome. Thus genetic typing of *H. pylori* isolates can shed the light on the severity of the disease. Since the cagA gene plays a very important role in such diseases, it was the most extensively studied. Comparative sequence analysis of cagA gene of isolates from patients with different gastric pathology, ethnic groups and geographical locations seems to be a method of choice in this regard. Furthermore no such analysis has been reported in Turkey yet. In this study we attempted to assess the genetic relationship between our isolates and those from other locations based on the *cagA* 5′ region sequences.

The sequence diversities of the cagA gene have been used to deduce the genetic relationships among isolates from Europe, USA, Asia and Africa [18,19]. Albert et al. [20] attempted to analyze the cagA genes of 9 isolates from ethnic Arab Kuwaiti patients and reported that isolates were closely related to the Indo-European group but distinct from East Asian strains. Cortes et al. [21] studied the cagA gene of isolates from 19 Philippinos and reported that the majority of those isolates were of the Western type suggesting Western influence. Rahman et al. [22] also did phylogenetic analysis using a 219 bp fragment near the 5′ end of cagA gene and showed that Bangladeshi isolates were more closely related to the Indian and Western isolates than those from Chinese and Japanese (East Asian) isolates. van der Ende et al. [6] also showed earlier that *H. pylori* cagA-positive isolates from China and Netherlands were distinct.

Based on the assumption of monophyletic origin of cagA sequences we can infer that our *H. pylori* isolates from the Anatolian region were distributed into two main groups as shown in our constructed phylogenetic tree the mixed group of African/Anatolian/Asian/European and the Anatolian/European sub-groups. The fact that Anatolian location connects the three continents Asia, Africa and Europe made it possible to suggest that *H. pylori* strains in those regions have undergone genetic modifications through migration events within those continents. Actually this has been shown on our phylogenetic tree by spotting location of Anatolian origin sequence clusters as one of those clusters was closely related to a distinct European group while the other was embedded into the mixed group which is close to the Asian cluster.

Although there is no particular cagA conserved region that could differentiate our Turkish isolates from the other isolates, our constructed phylogenetic tree showed that they were clustered based on the clinical outcome. We have found that our Turkish isolates from gastritis patients were clustered into 2 groups one that included the majority of the isolates and the other included the rest of the gastritis isolates together with isolates from peptic ulcer patients. Isolates from the latter groups were found to share several amino acids that were not found in the first group of gastritis alone. This might suggest that the progression of the disease from gastritis to peptic ulceration might be the result of frequent mutations that occur during the years of infection. The detection of conserved domains specific to those groups could be the reflection of association of those isolates with the clinical disease. In addition some amino acids were found only in peptic ulcer isolates but not in the gastritis isolates which might indicates that such isolates undergo further mutations. Nevertheless further studies comprising additional 5′ region sequences of cagA including isolates from cancer patients can provide more evidence on such correlation. In this study we have shown that our isolates were closely related to the European isolates although some have a tendency to form a separate cluster close to the mixed group. This shows that our isolates have a mixed gene pool while the others were much more scattered. Since the cagA gene undergoes selective pressure over the years a study that utilizes house-keeping genes will further confirm these results.

## Conclusions

This study provided an additional insight into the profile of our cagA gene which implies a relationship in geographic locations of the isolates.

## Methods

### H. pylori

Gastric antral biopsies collected from 19 Turkish patients (G = 12, DU = 5, GU = 2) were homogenized, inoculated onto Columbia agar plates with 5% horse blood and incubated at 37°C for 4–5 days under microaerophilic conditions.

### PCR

Genomic DNA extraction was done using the QIAamp DNA Mini Kit (QIAGEN, Germany) according to the manufacturer′s instructions. Amplification of the cagA 5′ conserved region was done using the primers F1 (5′-GATAACAGGCAAGCTTTTTGAGG-3′) and B1 (5′-CTGCAAAAG ATTGTTTGGCAG-3′) [23]. The amplification steps were done under the following conditions: 94°C for 1 min; 35 cycles of 94°C for 1 min and 57°C for 1 min, and 68°C for 1 min. Final extension was done at 68°C for 5 min. The mixture was stored at 4°C. PCR products were separated by 2% agarose gel electrophoresis and examined under UV illumination. The PCR products were then purified using QIAquick PCR purification kit (QIAGEN, Germany) according to the manufacturer’s instructions.

### DNA sequencing

DNA sequencing was performed by the BigDye Terminator v.1.1 Cycle Sequencing Kit in our lab using ABI PRISM 310 Genetic Analyzer (Applied Biosystems, USA).

### Phylogenetic analysis

The analysis included cagA 5′ region amino acid sequences of 52 isolates of which 19 were Turkish isolates and 33 from other countries including 2 reference strain sequences retrieved from GenBank. To clarify the phylogenetic relationship between our Turkish *H. pylori* isolates and those from other countries the cagA 5′ region was sequenced and then translated into amino acid sequence using the Open Reading Frame (ORF) Finder program. All possible transcripts of sequences were checked with BLAST program to determine the correct amino acids sequences. The translated 5′ region sequences of Turkish isolates were then aligned with other representative sequences from European, African, Asian and American-Indian isolates using multiple alignments with MUSCLE program [24]. Selection of the conserved blocks and removal of ambiguously aligned divergent blocks from alignment was done with Gblocks 0.91b software [25,26]. The alignment result was then subjected to PhyML 3.0 program [27] hosted on Web Server “Phylogeny.fr” [28] to build phylogenetic tree using Maximum likelihood algorithm [29]. Since the approximate likelihood ratio test (aLRT) and Shimodaira-Hasegawa (SH) provide a compelling alternative to conventional methods [30], phylogenetic tree was constructed using the aLRT/SH-like. The web based program Boxshade [31] was used for highlighting sequences according to the similarities. The non-Turkish sequences and their initials with GeneBank accession numbers used in the tree construction were the followings:

***hpEurope_1***:gi|306480322|, ***hpEurope_2***:gi|306480290|, ***hpEurope_3***:gi|306479848|, ***hpEurope_4***:gi|306479659|, ***hpEurope_5***:gi|306479690|, ***hpEurope_6***:gi|394913|, ***hpEurope_Swe***:gi|37811871|, ***hpEastAsia_hspEAsia_1***:gi|306480475|, ***hpEastAsia_hspEAsia_2:*** gi|306480413|, ***hpEastAsia_hspEAsia_3***:gi|306480260|, ***hpEastAsia_hspEAsia_4***:gi|306480140|, ***hpEastAsia_hspEAsia_5***:gi|306479818|, ***hpEastAsia_hspMaori_1***:gi|306480200|, ***hpEastAsia_hspMaori_2***:gi|306479989|, ***hpEastAsia_hspMaori_3***:gi|306479960|, ***hpEastAsia_hspAmerind_1***:gi|306480508|, ***hpEastAsia_hspAmerind_2***:gi|306480494|, ***hpEastAsia_hspAmerind_3***:gi|306479932|, ***hpEastAsia_hspAmerind_4***:gi|306479916|, ***hpEastAsia_hspAmerind_5***:gi|306479895|, ***hpAfrica1_hspWAfrica_1***:gi|306480170|, ***hpAfrica1_hspWAfrica_2***:gi|306480230|, ***hpAfrica1_hspWAfrica_3***:gi|306479787|, ***hpAfrica1_hspSAfrica_2***:gi|306479721|, ***hpNEAfrica_1***:gi|306480445|, ***hpSahul***:gi|306480354| ***,hpAsiaII_6:*** gi|306480019|, ***hpAsiaII_4***:gi|306480049|, ***hpAsiaII_3***:gi|306480079|, ***hpAsiaII_1***:gi|306480384|, ***hpAsiaII_5***:gi|306479878|, ***STR_26695***:gi|306479659|, ***STR_J99***:gi|4155035|.

## Competing interests

The authors declare that they have no competing interests.

## Authors’ contributions

BAS developed the idea, designed the methodology, analyzed the results and drafted the manuscript. SA carried out endoscopies, biopsy collection and patient evaluation. BKB grew the organism, PCR amplification and DNA sequencing of the gene. MTY carried out detailed analysis of the phylogenetic tree. All authors read and approved the final manuscript.
